# Laser Powder Bed Fusion of Metal Coated Copper Powders

**DOI:** 10.3390/ma13163493

**Published:** 2020-08-07

**Authors:** Viktor Lindström, Oleksii Liashenko, Kai Zweiacker, Serhii Derevianko, Vladyslav Morozovych, Yurij Lyashenko, Christian Leinenbach

**Affiliations:** 1 Empa-Swiss Federal Laboratories for Materials Science and Technology, Überlandstrasse 129, CH-8600 Dübendorf, Switzerland; viktor.lindstroem@empa.ch (V.L.); oleksii.liashenko@cdu.edu.ua (O.L.); kai.zweiacker@empa.ch (K.Z.); 2 Department of Physics, Cherkasy National University, 18000 Cherkasy, Ukraine; serhii.derevianko@cdu.edu.ua (S.D.); vladyslav.morozovych@cdu.edu.ua (V.M.); yurij.lyashenko@cdu.edu.ua (Y.L.)

**Keywords:** laser powder bed fusion, Cu-Sn, Cu-Ni, coated powder, in-situ alloying, balling defects

## Abstract

Laser powder bed fusion (L-PBF) of copper alloys with high copper content is difficult due to the high infrared reflectivity and thermal conductivity of these alloys. In this study a simple and scalable method for coating copper powder with tin and nickel is presented, and suggested as an alloying strategy for such alloys. The coated powders were processed in a commercial L-PBF-machine at various scanning speeds. The samples made from coated powders show a lower amount of porosity compared to samples made from in-situ alloyed powders of similar composition.

## 1. Introduction

Laser powder bed fusion (L-PBF) is a type of additive manufacturing (AM) where a thin layer of powder is deposited on a baseplate and a pattern molten by a laser. New layers are added and the melting is repeated, forming a 3D structure layer-by-layer [1,2]. This technology has attracted much attention in the scientific community for the possibility to manufacture part geometries impossible to manufacture using conventional processing methods [3]. It is also possible to produce materials with spatially varying microstructures, or including metastable phases [4,5]. L-PBF has become an industrially relevant method for manufacturing small batches or individualized parts quickly [6].

For more than 15 years, there has been an interest in manufacturing parts from copper [1] and precious metals like gold and silver alloys [2] using L-PBF. Target applications include heat exchangers, induction [3] and electric motor [4] coils, as well as radio frequency cathodes [3]. The manufacturing of dense, defect-free parts is, however, challenging due to the high thermal conductivity (400 W/mK) and high optical reflectivity (*R*), the complement of absorptivity (*A*)R=1−A, in the near infrared spectra (>99%) of copper. This means that most of the energy put into the system is either reflected or quickly dissipated away [5].

When processing copper powder at laser powers available in many commercial L-PBF machines, typically around 200–400 W, the parts are characterized by a relatively high level of porosity [6,7]. These porosities are often a result of balling [8], a defect characterized by the powder agglomerating into balls or tubes with a diameter larger than the powder diameter along the laser track. These defects are formed as the input power is too low for the molten powder to wet the underlying substrate.

To improve the quality of copper parts during L-PBF, several different approaches have been attempted:

1. High power lasers, on the order of 800 W, can be used to achieve dense samples [9,10,11]. Operating at this high power, however, comes with a high risk of damage to the equipment [11] due to back reflection of the intense laser light.
2. Dense samples can be achieved at lower power by using copper alloys with lower thermal conductivity. Copper alloys that have been successfully processed by L-PBF include Cu-Sn systems [12,13,14], CuCr and CuCrZr alloys [15] and CuNiSi alloys [4]. However, these alloys are less useful for many applications where high thermal conductivity is required.
3. Laser sources that are not of the conventional near-infrared type were also used for processing copper alloys. In this regard, green lasers with a wavelength of 532 nm were primarily used. In comparison with infrared light, copper has a significantly higher absorptivity in the green part of the spectrum. Green lasers with sufficient power and beam quality have only become commercially available the last years. Commercial machines using green laser sources have been advertised [16], but scientific literature is scarce on the topic. Direct metal deposition (DMD), an AM technique similar to L-PBF, as well as laser welding of copper with a green laser source have been reported in the scientific literature [5,17,18]. Green lasers have also been used in combination with infrared lasers in laser welding processes [19,20] where the green laser helped stabilizing the process. However, green lasers are still significantly more expensive than infrared lasers, and due to most metals having a relatively flat absorptivity curve in the visible and near-visible range, it can be expected that only highly specialized machines will be equipped with these sources in the near future.

Another type of laser source that has been used for L-PBF of copper is lasers with ultrafast pulsing [21]. This approach yielded cohesive parts at only 20 W of power, but the manufactured parts exhibit a high porosity as the powder particles are sintered together, rather than fused by melting. This indicates that significantly higher power is needed to form solid copper parts.

4. Recently, the L-PBF processability of copper has been increased by coating the surface of the powder with materials with a higher absorptivity. This has been achieved by oxidizing the copper surface [22], yielding decreased porosity, which agrees with similar results on 18 K gold alloys [23]. Mixing of copper powder with carbon nanoparticles has also been attempted [24], but did not have a significant effect on the porosity. In the latter case, it was speculated that the poor wetting of the carbon particles of copper was the reason for not seeing any improvement.

The use of coatings is interesting for applications where a specific alloy composition is desired. By coating a base powder rather than alloying, a higher absorptivity can be achieved, widening the processing window to allow processing at higher scanning speeds, or using a cheaper laser source with lower output powers. Further possible applications of coatings are to protect a sensitive base powder from oxidation, changing the wetting properties or to scan different alloy compositions.

In this work, we present a method for coating copper with thin layers of tin or nickel using an immersion deposition method. Similar methods are well known for tin [25,26] and nickel [27] coating of copper. These methods are based on the redox reaction

(1) $$Cu+X^{2+}\;↔Cu^{2+}+X$$

combined with a complexation reaction with thiourea, which although very complex, [28] is assumed to be well approximated by the reaction

(2) $$Cu^{2+}+4SC{\left(NH_{2}\right)}_{2}↔{\left[Cu{\left(SC{\left(NH_{2}\right)}_{2}\right)}_{4}\right]}^{2+}.$$

The equilibrium of (2) is shifted strongly to the right side, and thus allows the reaction to proceed.

The coating metals were chosen as they are used in many commercial copper alloys and are thus well studied and of industrial interest, as well as being electrochemically suitable for immersion deposition on copper. The method of coating is easily scalable and cost efficient and is expected to be transferable to other coatings and base materials such as silver and gold alloys, etcetera. The coated powders were consolidated by L-PBF using a commercial machine with relatively low maximum power (200 W) and investigated using X-ray computer micro-tomography (CT) and metallographic cross-sections with regard to their porosity.

## 2. Materials and Methods

### 2.1. Powder Coating

Copper powder was coated with tin and nickel using an immersion deposition method. Two separate preparation methods for coating the powders were used. In preparation method 1, gas atomized high conductivity oxygen free copper powder with a size of −45 to +20 µm (99.95%, Sandvik Osprey, Neath, UK.) was added to a heated solution of deionized water containing thiourea (99%, Alfa Aesar, Ward Hill, MA, USA.) and hydrochloric acid (Sigma Aldrich, St. Louis, MO, USA.). The solution was stirred so the copper particles where suspended, at which point a solution of tin(II) chloride (99%, EMD Millipore, Burlington, MA, USA.) or nickel(II) chloride (99%, Alfa Aesar, Ward Hill, MA, USA) was added. In preparation method 2 the same powder was used. In this case, the thiourea and tin(II) chloride were of unknown origin. The acid was either hydrochloric acid, or sulphuric acid as outlined in Table 1. In addition, sodium chloride was added to a concentration of 80 g/L. In this method the coating was performed twice on each batch of powder.

The coating reactions are run with the coating salt in excess, such that the metal deposition is limited by the point at which the powder surface is completely covered with either Sn or Ni. Hydrochloric acid deoxidizes the surface of the copper as well as protecting the oxidation sensitive Tin(II) chloride from oxidizing. It is therefore used at a higher concentration in the tin coating baths. The thiourea concentration is selected to be higher in the nickel coating baths as nickel has a lower electrochemical potential, and thus needs stronger complexation. The details of the coating baths are shown in Table 1. The batches are named after the coating metal and composition by weight after L-PBF processing.

Diffuse reflectance spectra of the powders were measured in Jasco V770 spectrophotometer (Jasco, Easton, MD, USA.) using powder holder (PSH-002, Jasco) in the back of the integrating sphere ISN-923 (Jasco). A reflectance measurement adapter was applied during the measurement to remove specularly reflected light component. The double reference method was used to recalculate the powder reflectivity with BaSO4 powder used for a baseline correction and acetylene carbon black powder used as a dark reference, all the signal corrections was made automatically in the Jasco software. The powder morphology was investigated by scanning electron microscopy (Nova NanoSEM 230, FEI, Hillsboro, OR, USA.) of both powder cross-sections and whole powder particles.

### 2.2. Laser Powder Bed Fusion

Cylinders with a height of 5 mm and diameter of 5 mm as well as cubes 5 × 5 × 5 mm^3^ were fabricated in a Sisma MySint 100 L-PBF machine (SISMA Piovene Rocchette Vicenza, Italy) equipped with a 200 W 1064 nm Nd:YAG laser with a Gaussian spot size (1/e^2^) of 55 µm. Reference samples were made from pure copper powder, bronze powder (Cu-7Sn-0.3Ni, wt.%), and mixtures of these with 50% and 25% bronze by weight. The laser power, P, was kept constant at 175 W, hatch distance, h, at 100 µm and layer thickness, l, at 40 µm. The scanning speed was varied between 25 and 900 mm/s. This corresponds to a volumetric energy density, defined as $E_{V}=\frac{P}{vhl}$, of 1800–49 J/mm^3^, respectively. Cubes and cylinders from the coated powders were manufactured using the same parameters as the baseline powders, except for the powder Coated Sn8.5, which was processed at 200 W. The baseplate was made from a copper alloy (Cu-4Zn-1Al, wt.%) and has a diameter of 34 mm.

As the powders Coated Sn 3.0 and Coated Ni 1.9 were used up for the experiments described above, an additional sample was manufactured from the batch Coated Ni 2.4. This 5 × 5 × 5 mm^3^ large cubical sample was processed on a stainless steel baseplate at 200 W with 100 mm/s scanning speed. The hatch was 100 µm and the layer thickness 20 µm.

### 2.3. Sample Analysis

The relative densities of the samples were determined from optical cross-sections and X-ray computer tomography. To avoid closing pores by smearing during sample preparation, cross-sections were carefully prepared by 3 µm diamond polishing followed by 50 nm silica particle polishing and vibration polishing using 50 nm alumina particles. The cross-sections were imaged in a Leica Axiovert 100 (Leica, Wetzlar, Germany) optical microscope as a 2.1 mm × 2.8 mm rectangle taken at the center of the cross-section; the image was binarized and thresholded in the ImageJ distribution FIJI [29] using the default thresholding method. X-ray tomography was performed on an EasyTom XL Ultra from RX Systems in a cone-beam setup with two sources (160 and 230 kV) with a 3-megapixel flat panel detector (Varian PaxScan, Palo Alto, CA, USA.). The reconstructed image was binarized and filtered using a 2 pixel × 2 pixel × 2 pixel median filter followed by thresholding in ImageJ using the Huang’s method [30] or the max entropy method [31] based on inspection of which gave better results. The relative density was calculated by counting pixels of a cube placed cut out from the center of the images.

The atomic composition after sample consolidation was measured from polished cross-sections by X-ray fluorescence in a Fischerscope X-Ray XDV-SDD (Fisher Technology Inc., Windsor, CT, USA). The phase composition was analyzed by X-ray diffraction in a Bruker R8 (Bruker, Billerica, MA, USA).

## 3. Results

### 3.1. The Manufactured Powders

**Figure 1:**
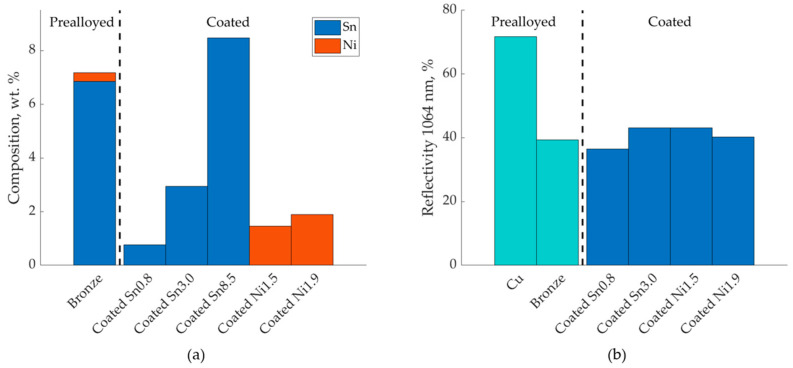
(**a**) Composition of the coated powders after L-PBF processing measured by X-ray fluorescence of cross-sectioned and polished samples and (**b**) optical reflectivity of the powder bed at 1064 nm for coated and non-coated powders.

**Figure 2:**
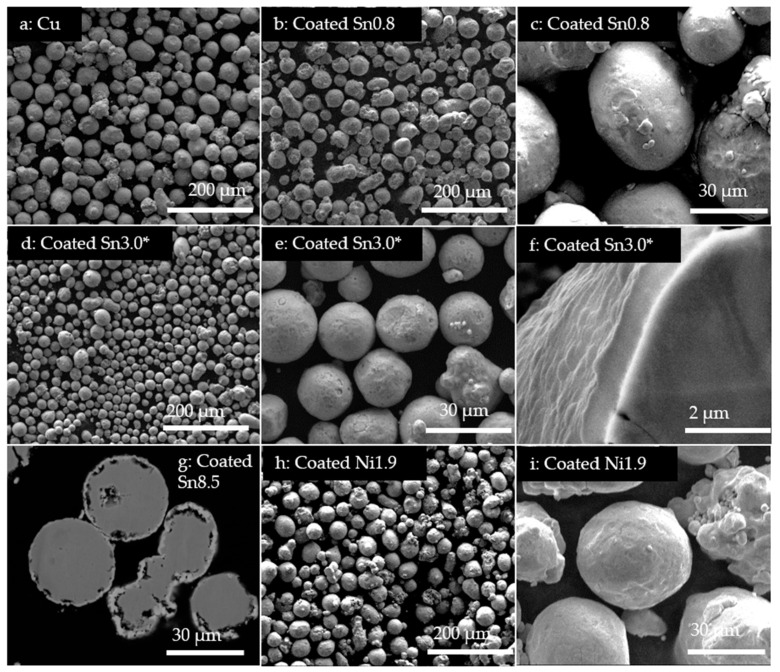
SEM images of the coated powders. (**a**) Pure Cu powder; (**b**–**c**) Coated Sn0.8 and (**d**–**f**) Coated Sn 3.0*, a batch of powder produced with the same method as Coated Sn.3.0. The coatings of the two powders are comparable in thickness, and morphology.; (**g**) cross section of Coated Sn8.5, with pores below the coating and (**h**–**i**) Coated Ni1.9.

The nominal compositions of the samples after L-PBF are shown in Figure 1 together with the measured optical reflectivities of the powders. The coated powders had optical reflectivities similar to that of the bronze powder, approximately 40%, whereas the copper powder had a significantly higher reflectivity of around 70%. The flowability of all powders was good, corresponding to between 1 (very good flowability) and 2 (sufficient flowability) on the optical evaluation method proposed by Spierings et al. [32], and a smooth powder layer was achieved upon deposition on the build plate. SEM images of the coated powders are shown in Figure 2. The powders Coated Sn0.8, Coated Ni1.5 and Coated Ni1.9, all had a smooth coating, which uniformly covered the copper particles. Coated Sn3.0 had a rougher surface, and Coated Sn8.5 even rougher with voids forming under the coating. It was observed that the morphology of the surface could be modified by changing the chemical environment in the coating, see the supplementary material, Figure S1 and Table S1.

### 3.2. Relative Densities and Microstructure Analysis of L-PBF Samples

**Figure 3:**
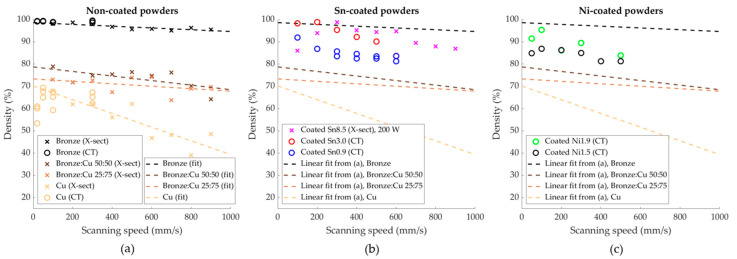
Relative densities of printed samples as measured by computer tomography (o) and metallographic cross-sections (x). The dashed lines are linear fits of the density of the uncoated powders to serve as a common visual guide in all three graphs, without implying any underlying physics. (**a**) Non-coated and blended powders; (**b**) Sn-coated powders and (**c**) Ni-coated powders. Note that the porosity of Coated Sn8.5 was measured by optical cross-sections, and was processed at a higher power than the other samples.

The measured relative densities of the printed samples are plotted in Figure 3. The relative densities of the printed reference powders of copper, bronze and blends thereof, are plotted in Figure 3a. Linear fitting curves of the density of the samples made from the uncoated powders are added in all the figures to serve as a visual guide without any physical significance. The porosity was decreasing with an increasing amount of tin, but did not vary significantly with changing scanning speed. The same trends were apparent with the Sn-coated powders. However, these samples had lower porosity than samples with comparable chemical compositions fabricated from powder blends.

**Figure 4:**
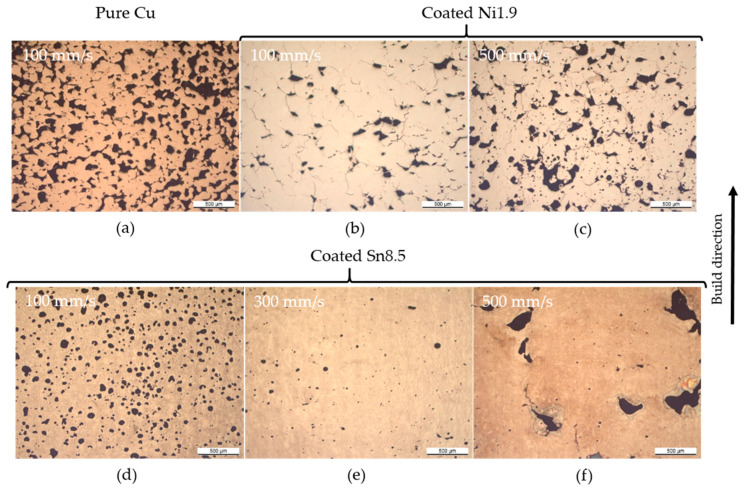
Optical cross-sections of samples printed from coated powders and pure copper. (**a**) Uncoated pure copper processed at 175 W where the balling type porosities are evident. (**b**,**c**) Coated Ni1.9 where the porosity is reduced with respect to pure copper. (**d**,**e**) Coated Sn8.5 processed at 200 W, with spherical pores in the high energy density regime, large irregular pores in the low energy density regime and a relatively dense sample in the middle.

**Figure 5:**
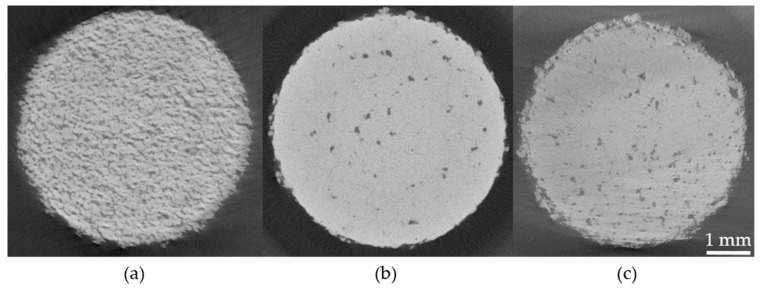
X-ray computer tomograms of (**a**) pure copper cylinder, (**b**) Coated Sn3.0 and (**c**) Coated Ni1.9. The cracks in the samples made from coated powder are visible and no loose powder is found in the pores. All tomograms are from samples produced at 100 mm/s scanning speed.

The pores in the samples have varying morphology across the different powders used, as shown in the optical cross-sections in Figure 4. The copper samples clearly exhibited balling porosity, and with increasing content of tin and nickel, respectively, these pores gradually disappeared. For the powder with the thickest coating, Coated Sn8.5, the pore morphology was dependent on the scanning speed, with low scanning speed leading to a high degree of spherical pores, and higher scanning speed leading to the appearance of large irregular pores. In-between these extremes an operating window leading to low porosity levels could be found. The balling porosity was also clearly visible in the tomograms of pure copper, see Figure 5 where the pores were large and well connected with the metal aggregated into connected blobs. It is noteworthy that no loose powder was found inside the large pores, which is a strong indication for balling. In the tomograms of Coated Sn3.0 and Coated Ni1.9, the individual pores were of approximately the same size, but much fewer and not connected.

**Figure 6:**
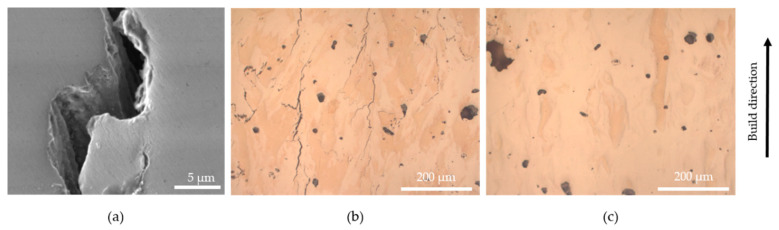
(**a**) SEM image of crack in Coated Sn3.0, showing dendrites typical of solidification cracking. (**b**) Metallographic cross-section of upper part of a sample made from Coated Ni2.4 on a preheated stainless steel baseplate, showing many cracks. (**c**) Same sample as in (**b**) in the lower part of the sample, without cracks.

Cracks were observed in the samples fabricated from CoatedSn3.0 and the nickel coated powders, see Figure 6a,b. These are supposed to be solidification cracks based on their morphology with dendrites in the crack surface, as shown in Figure 6a. In order to study the cracking a further sample from the powder Coated Ni2.4 was fabricated on a stainless steel baseplate. In this case, the baseplate was scanned with the laser as a grid with 1 mm spacing at 500 mm/s and 100 W laser power before the deposition of powder. These parameters were selected to achieve high energy density leading to a hotter base plate in order to reduce thermal stresses and thus crack formation in the L-PBF process, although the temperature of the base plate was not measured. As can be seen in Figure 6c, the number of cracks could be reduced in the lower part of the sample, but many large, up to 1 mm long cracks were observed in the upper part of the sample.

## 4. Discussion

The coating method presented above yields a smooth and complete coating of the powder. At the same time, it is simple, cost efficient and scalable. The powders retain good flowability after the coating, which is important for L-PBF applications. One possible issue for scaling the process is that the coatings are sensitive to the process conditions. The sensitivity is well illustrated by the two nickel coatings, which were produced identically, except for the rate of addition of the salt. The reason for this is assumed to be that the concentration of the ions influences the nucleation of the coating on the surface. It is known from electrodeposition that the nucleation density is higher with lower ion concentration [33,34], and similar mechanisms should lead to similar results for immersion depositions as well. The more nucleation sites on the surface, the smoother and thinner the coating can be expected. Another relevant factor is the presence of impurities, which have been identified to be important for the smoothness of immersion deposition coatings [35]. Impurities might be the reason for the significantly thicker coatings on the powders produced according to preparation method 2. However, many other parameters, like temperature, pH, potential difference and impurities are highly significant. However, a detailed study of the influence of these factors on the coating quality is beyond the scope of the present work.

The comparison of the samples fabricated from coated, non-coated powders and the powder blends show that coating powders with a thin metal coating of tin decreases the porosity of the built samples, in comparison with the non-coated powder blends of similar compositions. The nickel-coated copper powders achieve a similar relative density as the tin-coated powders. It can be assumed that the achieved density is also better than what would have been achieved with prealloyed alloys of similar composition since nickel, in copper at the relevant compositions, behaves similarly to tin. Both elements form solid solutions, which decrease thermal conductivity and optical reflectivity. The main difference with nickel as a coating is that the melting point, unlike for tin, is higher than that of copper. However, this is assumed to be beneficial for the consolidation process as the coating can be expected to survive longer due to a later onset of the melting.

**Figure 7:**
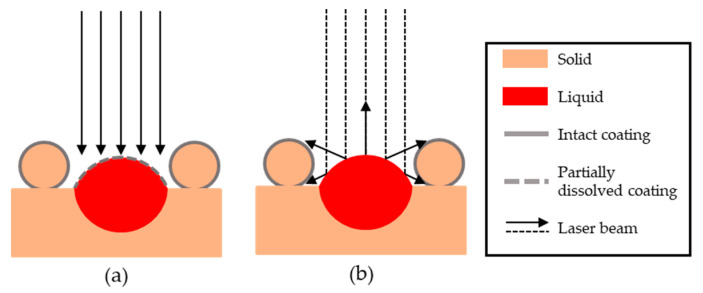
Possible mechanisms for the increased coupling of coated powders: (**a**) Coating survives, at least partially, long enough to form a deep weld and (**b**) surrounding powder gets heated by secondary reflections from the melt pool.

The reduced porosity is assumed to be due to the increased surface absorption from the powder relative to non-coated powders and powder blends of similar composition. This increased absorptivity will increase the overall heat input, widening the melt pool, leading to the transition to conduction mode melting. Any such effect will only be present until the tin has dissolved and homogenously distributed in the copper matrix. A possible secondary effect is that reflected light will be better absorbed in the surrounding powder. This increased absorption can lead to a preheating of the powder, which would lead to an increase of the effective power of the laser by a factor $T_{M}/\left(T_{M}-T_{0}\right)$, with $T_{m}$ being the melting temperature and $T_{0}$ the substrate temperature, both relative to an unheated substrate [36]. These hypothesized mechanisms are illustrated schematically in Figure 7.

The measured relative densities of the samples had, except for Coated Sn8.5, a low dependence on the scanning speed. This can be qualitatively understood from the high thermal conductivity of these alloys, making the conductive heat transfer the dominating term in the heat balance of the substrate. This in turn results in a semispherical melt pool [36]. The melt-pool dimensions and the temperature distribution in the melt pool are only weakly dependent on the laser scanning speed. The Péclet number (sometimes referred to as normalized traverse rate) is defined as:

(3) $$Pe=\frac{v\cdot a}{\alpha },$$

with  v the scanning speed, a the 1/e^2^ spot radius and α the thermal diffusivity. In order to achieve a high scanning speed dependence a Peclet number *Pe* > 1 needs to be reached. In the case of copper, this would require scanning speeds significantly above 5 m/s. This means that, considering a fixed laser spot size, the laser power is the main factor determining the properties of the melt pool. The importance of the power relative to scanning speed has already been shown experimentally in a previous study [11]. However, this correlation indicates that thin powder coatings might be an interesting strategy for improving the quality of parts fabricated from materials like Cu or Au, as it increases the coupled power of the powder bed, leading to a wider processing window for a given powder composition.

Cracking of gold alloys with low amounts of additional alloying elements have been previously reported in the literature [37]. These alloys have similar physical properties to the copper alloys studied in this paper, so cracking seems to be a general concern when alloying this class of materials. It is believed that the cracking observed in this study can be significantly reduced by process optimization. This is supported by the decreased crack density in the lower part of the sample fabricated from Coated Ni2.4, which is presumably due to the higher temperature close to the preheated stainless steel base plate resulting in lower residual stresses. Possible techniques to reduce cracking include preheating [38], wobbling of the laser scan head [39] and other methods of controlling the residual stress. A systematic study of these approaches was not in the scope of this publication and will be part of future work.

## 5. Conclusions

In this work, copper powder was coated with thin layers of tin and nickel using an immersion deposition method. Small samples were manufactured from this powder and compared with samples fabricated from copper and mixtures of pure copper powder and bronze (Cu–7 Sn–0.3 Ni, wt.%). These samples were analyzed with regard to porosity, as measured by computer tomography and microscopy of metallographic cross-sections. The samples made from tin-coated copper have a significantly lower porosity than the powder mixtures of similar composition. The nickel-coated powders yield parts with a similar density to the tin-coated powders. The reduced porosity is assumed to be due to the transition from the balling regime to the conduction welding regime because of the increased optical absorption of the powder bed.

## Figures and Tables

**Table 1:** Summary of immersion deposition solutions for the coated powders used in the study.

Powder Designation	Coating (salt)	Powder Mass (g)	Solution Volume (L)	Reaction Temp. (°C)	Reaction Time (min)	Concentration Thiourea (M)	Concentration Acid (M)	Concentration Cation (mM)	Addition of Salt Solution	Preparation Method
Coated Sn0.8	Sn (SnCl_2_)	103.86	0.8	43	1.5	0.41	0.40 (HCl)	76	All at once	1
Coated Sn3.0	Sn (SnCl_2_)	100	1.0	64	20	0.66	0.31 (H_2_SO_4_)	21	All at once	2
Coated Sn8.5	Sn (SnCl_2_)	25	1.0	60	20	1.1	0.29 (HCl)	21	All at once	2
Coated Ni1.5	Ni (NiCl_2_)	120.44	0.8	52	2.5	1.64	0.12 (HCl)	70	All at once	1
Coated Ni1.9	Ni (NiCl_2_)	120.47	0.8	50	2.5	1.64	0.08 (HCl)	70	Dropwise	1
Coated Ni2.4	Ni (NiCl_2_)	200.25	2.0	52	3	1.60	0.20	47	Dropwise	1

## Supplementary Materials

The following are available online at https://www.mdpi.com/1996-1944/13/16/3493/s1, Figure S1: Tuning the surface morphology of coated powders, Table S1: Recipes for powders in Figure S1.
