# Supplementary material for: Laser Powder Bed Fusion of Metal Coated Copper Powders
Source: Materials (Basel). 2020 Aug 7;13(16):3493. doi: 10.3390/ma13163493 (PMC7475881; doi:10.3390/ma13163493)
Supplement: Supplementary file 1 [file materials-13-03493-s001.pdf]

materials

Supplementary Material

Tuning the Surface Morphology of Coated Powders

It was noticed that the surface morphology could be tuned by altering the concentration of the reagent salt in the coating solution. At high concentrations of reagent the surface is smooth and covers the surface completely. At low concentration the surface is rough and islands of coating is formed. When the reagent salt was added dropwise, which should correspond to the lowest concentrations (assuming the reaction is fast on the time scale of the drop interval) the surface is covered by separated islands of coating.

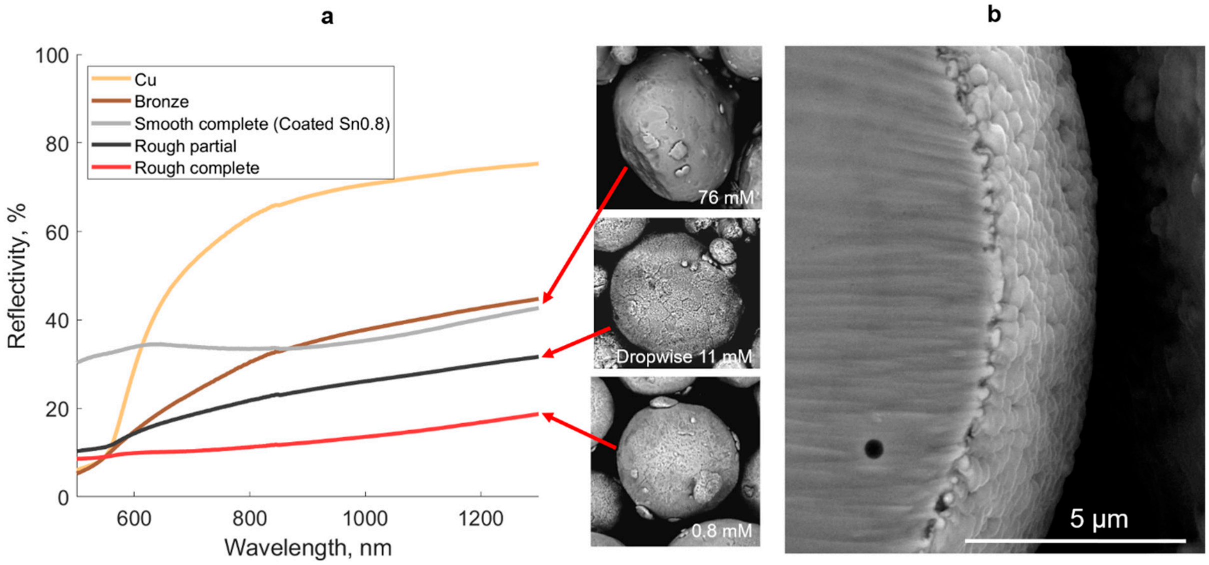

Figure S1: (a) The reflectivity of a powder bed of three tin coated powders with different surface morphology. (**b**) A cross-section of the powder named "Rough completed".

Table S1: The recipes for the powders in Figure S1, all being coated using "method 1".

Powder Designation	Coating (salt)	Powder Mass (g)	Solution Volume (L)	Reaction Temp. (°C)	Reaction Time (min)	Concentration Thiourea (M)	Concentration HCl (M)	Concentration Cation (mM)	Addition of Salt Solution
Coated Sn0.8	Sn ($SnCl_2$)	103.86	0.8	43	1.5	0.41	0.40	76	All at once
Rough partial	Sn ($SnCl_2$)	150.04	0.8	42	3	0.40	0.40	11	Dropwise over 3 minutes
Rough complete	Sn ($SnCl_2$)	10.00	0.5	40	short	0.34	0.42	0.8	All at once
